# Associations between parental bonding, social isolation and loneliness: do associations persist in later life and is isolation a mediator between parental bonding and loneliness?

**DOI:** 10.1186/s40359-022-00855-z

**Published:** 2022-06-16

**Authors:** Annette Burns, Gerard Leavey, Roger O’Sullivan

**Affiliations:** 1 Institute of Public Health, Dublin, D08 NH90 Ireland; 2 The Bamford Centre, Ulster University, Coleraine, BT52 1SA UK

**Keywords:** Loneliness, Parental Bonding, Social isolation, Middle age, Older adults

## Abstract

**Background:**

Poor parental bonding in childhood has been associated with loneliness in younger populations. Whether these associations persists into middle and older adulthood is unclear. Additionally, given the overlapping relationship between loneliness and social isolation we sought to explore the role of social isolation in any associations present i.e. are those reporting worse parental bonding lonely due to less connections or are they more likely to be lonely regardless of isolation.

**Methods:**

Analysis of a nationally representative longitudinal sample of adults aged 50 and over from the English Longitudinal Study of Ageing was undertaken. The current analysis was based on data for core participants across waves 3[2006/7] to 8[2016/17] with missing data across waves leading to analytical samples ranging from 4384 to 5173. Multivariate adjusted multinomial regression models were used to assess associations between parental bonding [PBI], isolation [score derived from data on living alone, frequency of contact with friends, family and children, and whether or not participate in social organisations] and loneliness [R-UCLA].

**Results:**

Parental bonding scores were associated with later life loneliness according to overall PBI score [RRR .93 95%CI .92-.95], care [RRR .90 95%CI .88-.92] and overprotection [RRR 1.11 95%CI 1.08–1.14] subscale scores as well as when separated into maternal and paternal scores, with effects larger in relation to chronic loneliness. Parental bonding scores were also associated with isolation in later life, with the exception of maternal overprotection which was non-significant. The addition of isolation to the loneliness models however had no impact on associations indicating that isolation is not a mediator of the association between parental bonding and later life loneliness.

**Conclusions:**

Associations between parental bonding and loneliness do persist into middle and older adulthood and were in line with hypothesis stronger for more chronic loneliness. Isolation did not explain these associations and those reporting more negative parental bonds were more likely to be lonely regardless of isolation.

**Supplementary Information:**

The online version contains supplementary material available at 10.1186/s40359-022-00855-z.

## Introduction

The terms loneliness and social isolation are two separate constructs, but are often used interchangeably, with loneliness sometimes considered an outcome of social isolation. However, loneliness is a very personal and subjective experience – it is the outcome of the difference between an individual’s preferred and actual experience of their social or emotional connections. Whereas social isolation is an objective measurement of the number of meaningful social relationships and social contact with others. Not everyone who is lonely is socially isolated and not everyone who is socially isolated is lonely. There are three main types of loneliness: social, emotional and existential as well as varying frequency. Social isolation on the other hand is focused on understanding the size of one’s network and frequency of interactions with one’s primary groups [family, friends, and neighbours] and secondary social groups [clubs, organisations, churches][1].

Studies in younger adults have demonstrated an association between parental bond and loneliness [2–4]. However, this remains unexplored in older adults with a lack of studies employing large population samples also. The impact of other early life circumstances, such as childhood socio-economic circumstances and parental substance abuse, on later life loneliness has recently been noted [5] however suggesting that an association between parental bonding and late life loneliness is also likely. Should such an association be present, it is likely indicative of more lifelong issues in relation to loneliness. Chronic or persistent loneliness has been highlighted as a particularly important group who are distinct and experience the greatest negative outcomes in terms of health outcomes [6]. It is therefore important to understand what proportion of loneliness in older adults might be linked to early life circumstance such as parental bonding, rather than late-life situational factors such as bereavement and isolation, and thus be a more chronic issue rather than more transient and situational loneliness. This is especially important from a policymakers and service providers’ perspective given the vastly different approaches to prevention and intervention which would be required. Too often loneliness interventions have been ineffective as they fail to address the root causes or have not been tailored to the needs of the individual. Rather than only focusing on loneliness in later life in isolation, we may need to recognise that by potentially addressing parental bonding in early years we can help avoid a lifetime of loneliness in some adults and especially in later life [7].

A growing body of academic interest and research has been devoted to the concept of attachment in childhood. As first described by Bowlby (1958) the theory suggests that when a child is able to establish a good attachment with a primary carer, often in a contained and supportive environment, this forms a secure base from which a child may safely explore his or her world [8]. This secure base is internalised and maintained as the person transitions into adulthood. Dissatisfaction with one’s connections [quality or quantity] may be linked to parental bonding and attachment [9, 10]. The development of a positive internal working model of the self and others is significantly related to the individual’s ability to make strong, satisfying relationships over the life-course [11, 12]. Thus, when an individual only experiences an insecure base in childhood, this is likely to create a sequalae of problematic and unstable relationships in adulthood in which low self-esteem and mistrust play a part. Additionally, previous research suggests that such people in later life who experience declining self-capacity may emphasise independence and self-reliance rather than interdependence [13]. Other evidence suggests that early attachment might influence the development of affective, cognitive, and neurobiological resources [14]. Thus, we can begin to articulate the relationship between early attachment experiences and later life loneliness.

From this, we were interested in exploring if people with poorer parental bonds make less connections and have fewer people in their lives or are they less satisfied with the connections they have and more likely to report loneliness regardless of isolation. It seems likely that those reporting negative parental bonding will be both more isolated and more lonely, with their isolation not fully explaining the loneliness they experience.

The aim of this study therefore was to investigate associations between parental bonding, loneliness and isolation among a longitudinal population representative sample of community-dwelling adults aged 50 years and older. We hypothesised: [1] Recalled parental bonding [overall, care and overprotection] will be associated with both isolation and loneliness in later life with overall and care scores displaying negative associations while overprotection scores will display positive associations. [2] Associations with more chronic loneliness will be stronger. [3] Associations between parental bonding and loneliness will be only partially mediated or explained by isolation.

## Methods

### Participants

The sample comprised participants from the English Longitudinal Study of Ageing (https://www.elsa-project.ac.uk/) which provides a nationally representative sample of community dwelling adults aged 50 and over living in England. ELSA began in 2002 with a nationally representative sample of 12,099 individuals aged 50 and over. The original sample was drawn from households that had previously responded to the Health Survey for England (HSE) and this is also used to refresh the sample periodically to ensure that it remains representative. Wave 3 (2006/7) includes 9,771 participants, 7,855 of whom completed a life history interview. Wave 8 (2016/17) included 8,445 participants [15]. In relation to the current analysis, for which wave 3 was the baseline and parental bonding indicators the exposure variable, follow up loneliness and isolation data from waves 4–8 was missing for 454 and 306 participants respectively. All those missing data for parental bonding in Wave 3 and loneliness or isolation in waves 4 to 8 were coded as missing in analytical models.

### Measures

#### Loneliness [outcome variable]

The outcome variable was loneliness as captured by the widely used 3-item short form UCLA Loneliness scale [16, 17] which asks respondents about how often they feel ‘left out’, ‘isolated from others’ of lacking in ‘companionship’. Each item was measured on a 3-point Likert scale with response options: ‘hardly ever/never’, ‘some of the time’, and ‘often’ resulting in a total score ranging between 3 and 9 with a higher score denoting greater loneliness. In line with previous analyses [18, 19], those scoring 6 and above were defined as lonely. For the current analysis loneliness data from waves 4 to 8 was included. Occurrences of loneliness were counted across waves 4 to 8 and a variable was generated group respondents by the number of waves for which loneliness was reported [i.e. no waves; one wave; two or more waves].

#### Parental bonding instrument [exposure variable]

The seven-item Parental Bonding Instrument [PBI] includes three care items [understood my problems and worries [reverse scored]; mother/father seemed emotionally cold to me; and she/he made me feel not wanted] and four overprotection items [let me do things I liked; liked me to make my own decisions; made me feel dependent on them [reverse scored]; and were overprotective of me [reverse scored] for each parent with a four-point response scale with responses ranging from ‘strongly agree’ to ‘strongly disagree’ [20, 21]. Higher scores on the care subscale indicated greater caring from parents and were therefore desirable while higher scores on the overprotection subscale indicate greater overprotection and are therefore undesirable.

Optimal parenting is characterised by high care and low overprotection scores. Thus, for the overall PBI score, care items were scored positively while overprotection items were scored negatively so that highest overall PBI scores indicate highest care and lowest overprotection scores. Items were summed to create an overall PBI score for both parents [ranging from 0 to 42] as well as an overall score for each parent separately [ranging from 0 to 21].

Subscales capturing overall care [ranging from 0 to 18] as well as for mother and father separately [ranging from 0 to 9] and overall overprotection [ranging from 0 to 24] as well as for mother and father separately [ranging from 0 to 12] were also created. Those who only responded for a single parent were included for mother or father scores as appropriate but excluded from overall parental scores.

#### Social isolation [potential mediator]

Social isolation was also adjusted for using a previously used ELSA approach [18, 22, 23] whereby one point was allocated for each of the following: living alone, having less than monthly contact with each of children, other members of the family and friends [in person/ telephone], and not being a member of organisations. Scores were summed to provide a social isolation index ranging from 0 to 5, which was then dichotomised, as in previous investigations, to distinguish between low [< 2] and high [≥ 2] levels of social isolation [18, 22, 23]. For the current analysis isolation data from waves 4 to 8 was included. Occurrences of high social isolation were counted across waves 4 to 8 and a variable was generated grouping respondents by the number of waves for which high isolation was reported (i.e. no waves; one wave; two or more waves).

### Covariates

Models were also adjusted for demographic variables [age, sex, education, marital status] when modelling isolation. In modelling loneliness other known and potential confounders which were significantly associated with loneliness (see Table 1) were also included [socioeconomic status [tenure and occupation at Wave 3]; and circumstances before aged 16 including severe financial hardship; either parent unemployed for more than 6 months when they wanted to be working; and parents drank excessively, took drugs or had mental health problems. Occupational class at W3 was measured using the National Statistics Socio-economic Classification and included 3 categories: managerial and professional occupations; intermediate occupations; and semi-routine and routine/manual occupations.

**Table 1:** Descriptive characteristics of ELSA cohort at wave 3 by loneliness [W4-W8]

	Lonely at no Waves		Lonely at 1 Wave		Lonely at 2 + Waves		F/χ2	*P* value
	n = 7,907		n = 1,985		n = 2,026			
	66%		17%		17%			
	n	%	N	%	n	%		
Sex [n = 6,510]								
Male	2044	48	437	42	423	34	79.4	< .001
Female	2192	52	601	58	813	66		
Age group [n = 7,383]								
50–54	646	14	183	15	216	16	87.7	< .001
55–59	1019	21	212	17	288	21		
60–64	868	18	194	16	247	18		
65–69	704	15	150	12	202	15		
70–74	688	14	160	13	171	12		
75–79	448	9	136	11	157	11		
80–84	265	6	104	9	89	6		
85 plus	135	3	76	6	25	2		
Education [n = 7,359]								
Degree	967	20	191	16	196	14	64.9	< .001
Intermediate	2640	56	620	51	787	57		
None	1150	24	398	33	410	29		
Marital status [n = 7,383]								
Married/Civil Partnership	3519	74	732	60	713	51	372.6	< .001
Cohabiting	237	5	39	3	48	3		
Single, Never Married	183	4	79	7	91	7		
Widowed	505	11	225	19	301	22		
Divorced	271	6	113	9	204	15		
Separated	58	1	27	2	38	3		
Occupation class [n = 7,260]								
Professional/Managerial	1767	38	344	29	386	28	71.6	< .001
Intermediate	1229	26	305	26	366	27		
Routine/Manual	1710	36	532	45	621	45		
Tenure [n = 7,379]								
Own outright	2916	61	694	57	768	55	19.1	< .001
Do not own outright	1855	39	520	43	626	45		
Either parent unemployed for more than 6 months when they wanted to be working when respondent < 16 [n = 5,532]	245	7	58	7	98	10	9.67	0.008
Parents drank excessively /took drugs/had mental health problems when respondent < 16 [n = 5,556]	179	5	63	7	89	9	22.7	< .001
Experienced severe financial hardship < 16 [n = 5,406]	65	2	20	2	40	4	16.6	< .001
Parental Bonding [Exposure]	M	[SD]	M	[SD]	M	[SD]		
Overall PBI [n = 4,612]	31	[5.61]	29.8	[5.95]	28.4	[6.34]	70.1	< .001
Overall care [n = 4,774]	13.8	[3.12]	13.3	[3.33]	12.5	[3.57]	59.8	< .001
Overall overprotection [n = 4,709]	6.85	[3.27]	7.59	[3.45]	8.18	[3.58]	59.1	< .001
Mother overall [n = 5,208]	15.6	[3.18]	15	[3.43]	14.3	[3.68]	63	< .001
Mother care [n = 5,311]	7.08	[1.76]	6.83	[1.91]	6.38	[2.15]	55.5	< .001
Mother overprotection [n = 5,283]	3.5	[1.89]	3.88	[2.00]	4.14	[2.06]	45.7	< .001
Father overall [n = 5,064]	15.3	[3.14]	14.6	[3.36]	14.1	[3.50]	63.7	< .001
Father care [n = 5,169]	6.7	[1.81]	6.43	[1.97]	6.07	[2.09]	41.9	< .001
Father overprotection [n = 5,129]	3.36	[1.81]	3.79	[1.90]	4.08	[2.00]	61.8	< .001
Social Isolation [W4-W8] [n = 10,605] [Potential Mediator]								
Never reported High isolation	3901	57	782	44	629	33	584	< .001
High isolation at one wave	1432	21%	467	26	346	18		
High isolation at 2 + waves	1570	23	533	30	945	49		

### Analysis

Key variables and demographic characteristics of the sample were compared according to reported loneliness using analysis of variance models and chi-square as appropriate. T-tests for significance were run to check for associations between parental bonding indicators and missing data for isolation and loneliness.

Multinomial logistic regression analysis was conducted to investigate the associations between indicators of Parental Bonding and Social Isolation [at one or multiple waves] with models adjusted for age, sex, education and marital status.

Multinomial logistic regression models were then employed to explore the associations between indicators of Parental Bonding and Loneliness [at one or multiple waves]. These models were adjusted for potential confounders including socio-demographic characteristics [age, sex, education, marital status] and additional potential confounders [[socioeconomic status [tenure and occupation at Wave 3]; and circumstances before aged 16 including severe financial hardship; either parent unemployed for more than 6 months when they wanted to be working; and parents drank excessively, took drugs or had mental health problems based on Wave 3 Life History Interviews].

Baron and Kenny's four step approach was employed to test for mediation [24]. Firstly, as above, we tested to see if the independent variable, indicators of PBI, predicted the dependent variable loneliness. Secondly, and also already encompassed in aim one, we tested to see if indicators of PBI predicted Social Isolation. Thirdly, it was assessed whether the potential mediator, Social Isolation, predicted Loneliness even while adjusting for indicators of PBI. Finally, Social Isolation was added to models predicting Loneliness and changes in the association between indicators of PBI and Loneliness were observed for mediation effects.

Data analysis was performed using Stata 16.0.

## Results

Among ELSA respondents aged 50 and over, 17% reported loneliness at a single wave and 17% reported loneliness at multiple waves. As shown in Table 1, reporting loneliness in mid/later life was associated with sex, age, education, marital status, social class in terms of occupation class and tenure, as well as difficult live events prior to age of 16 including parental unemployment, parental drug or alcohol misuse or mental health problems and severe financial hardship. Parental Bonding scores (as recalled at Wave 3) were also universally significantly associated with loneliness, with lower overall and care scores and greater overprotection scores associated with greater loneliness. Isolation and isolation at multiple waves also displayed a strong association with loneliness, with 49% of those reporting loneliness at multiple waves also reporting high isolation at multiple waves (χ2 = 584.0 *P* < 0.001).

Due to missing values analytic samples ranged from 4,384 to 5,173. Sample sizes for each model are included below [Tables 2–3].

**Table 2:** Multinomial models for social isolation [no waves/ one wave/ multiple waves] according to various indicators of parental bonding

Model		n	RRR	95% CI
1	Overall PBI	4753		
	Isolated at one wave		0.98	.96-.99***
	Isolated at multiple waves		0.97	.96-.99***
2	Overall Care	4923		
	Isolated at one wave		0.95	.93-.98***
	Isolated at multiple waves		0.95	.93-.97***
3	Overall Overprotection	4856		
	Isolated at one wave		1.03	1.01–1.05*
	Isolated at multiple waves		1.03	1.01–1.05*
4	Mother overall	5069		
	Isolated at one wave		0.97	.95-.99*
	Isolated at multiple waves		0.97	.95-.99*
5	Mother Care	5173		
	Isolated at one wave		0.94	.90-.98**
	Isolated at multiple waves		0.94	.90-.97**
6	Mother Overprotection	5145		
	Isolated at one wave		1.03	.99–1.07
	Isolated at multiple waves		1.02	.99–1.06
7	Father overall	4929		
	Isolated at one wave		0.95	.93-.98***
	Isolated at multiple waves		0.95	.93-.97***
8	Father Care	5036		
	Isolated at one wave		0.92	.89-.96***
	Isolated at multiple waves		0.91	.87-.94***
9	Father Overprotection	4989		
	Isolated at one wave		1.06	1.02–1.10**
	Isolated at multiple waves		1.05	1.02–1.10*

Adjusted for age, sex, education, marital status

**Table 3:** Multinomial models for loneliness [no waves/ one wave/ multiple waves] according to various indicators of parental bonding and with mediation analysis adjusting for social isolation

					Adjusted for Isolation		
Model		n	RRR	95% CI	n	Adjusted RRR	95% CI
1	Overall PBI	4384			4384		
	Lonely at one wave		0.97	.95–.98***		0.97	.95–.98***
	Lonely at multiple waves		0.93	.92–.95***		0.94	.92–.95***
2	Overall Care	4526			4526		
	Lonely at one wave		0.95	.93–.98***		0.95	.93–.98**
	Lonely at multiple waves		0.9	.88–.92***		0.9	.88–.92***
3	Overall Overprotection	4471			4471		
	Lonely at one wave		1.06	1.03–1.09***		1.06	1.03–1.09***
	Lonely at multiple waves		1.11	1.08–1.14***		1.11	1.08–1.14***
4	Mother overall	4889			4637		
	Lonely at one wave		0.96	.93–.98***		0.96	.93–.98**
	Lonely at multiple waves		0.9	.89–.92***		0.9	.88–.92***
5	Mother Care	4979			4724		
	Lonely at one wave		0.95	.90–.99*		0.94	.90–.99*
	Lonely at multiple waves		0.85	.82–.89***		0.85	.82–.89***
6	Mother Overprotection	4953			4699		
	Lonely at one wave		1.09	1.04–1.13***		1.08	1.04–1.13***
	Lonely at multiple waves		1.17	1.13–1.22***		1.18	1.13–1.22***
7	Father overall	4776			4526		
	Lonely at one wave		0.94	.91–.96***		0.94	.92–.97***
	Lonely at multiple waves		0.9	.88–.92***		0.9	.88–.92***
8	Father Care	4867			4613		
	Lonely at one wave		0.92	.88–.96***		0.92	.88–.97**
	Lonely at multiple waves		0.85	.82–.89***		0.86	.82–.90***
9	Father Overprotection	4832			4579		
	Lonely at one wave		1.12	1.07–1.17***		1.11	1.06–1.16***
	Lonely at multiple waves		1.19	1.14–1.24***		1.19	1.14–1.24***

Adjusted for age; sex; education; marital status; social class at W3 [tenure and occupation class]; either parent unemployed for more than 6 months when they wanted to be working when respondent under 16; parents drank excessively, took drugs or had mental health problems when respondent under 16; experienced severe financial hardship before 16

As shown in Table 2, Parental Bonding scores were significantly associated with reporting isolation at a single or multiple waves with all associations in the expected directions. The exception to this was overprotection scores for mothers only which were not significantly associated with isolation. Overall PBI scores and care scores (where a higher score indicates optimal parenting) displayed consistent significant negative associations with isolation [with relative risk ratios [RRRs] ranging from 0.98 to 0.92 for single wave isolation; and 0.97 to 0.91 for isolation at multiple waves. Overprotection scores meanwhile [where a higher score indicates less optimal parenting] displayed significant positive associations with isolation at multiple waves, in relation to parents overall as well as for fathers individually. Fathers also appeared to display the strongest association in relation to care, with an adjusted RRR of 0.91 (95%CI 0.87–0.94) for isolation at multiple waves.

PBI scores were also significantly associated with loneliness (Table 3). Associations were again all in the expected directions with associations also consistently stronger for loneliness at multiple waves. Higher PBI and Care scores were negatively associated with loneliness at both one and multiple waves with similar sized associations for mothers and fathers, while higher overprotection scores were positively associated with loneliness at both one and multiple waves, with very slightly larger associations seen for fathers.

As per Baron and Kenny's four steps for mediation, the independent variable, PBI, therefore predicted the dependent variable loneliness (Table 3, middle columns) [24]. As per Table 2, the independent variable, PBI, also predicted isolation. Further regression analyses confirmed that isolation predicted both single and multiple wave loneliness, with significant associations for multiple wave isolation when PBI was included (step 3). Finally in relation to step four, the addition of isolation to models (Table 3, right columns) had virtually no impact indicating that isolation was not a mediator of the association between PBI and loneliness [24].

## Discussion

In this population-based dataset of adults aged 50 and over in England, parental bonding was associated with both loneliness and isolation in later life and also had an association with loneliness independent of association. Those with worse parental bonding scores were more likely to report loneliness regardless of isolation. This suggests these individuals are not simply more isolated but are also less satisfied with the relationships they have.

We are not aware of a paper which has previously explored associations between parental bonding and social isolation. In the current study parental bonding scores were significantly associated with later life social isolation at either a single or multiple waves with a single exception in the case of mother overprotection for which associations were not significant in the current analysis. All associations were in the expected directions, with care and overall scores negatively associated with high isolation while overprotection was positively associated with high isolation. Across overall as well as care and overprotection subscales, fathers seemed to have a greater impact on later life social isolation in this sample compared to mothers.

In relation to loneliness, previous research has implied mothers are more impactful than fathers [with Robinson et al. finding greater maternal involvement was predictive of less social loneliness in young adults] [25]. Our findings did not support this however as in line with our findings in relation to isolation, fathers appeared as impactful as mothers and sometimes slightly more so. Overall though the consistent associations between parental bonding across parents with loneliness in later adulthood supports and extends the findings of previous researchers who found a consistent connection with parental care which while decreasing in importance with age remained relevant to loneliness at age 31 [3]. Von Soest et al. suggested that the ongoing importance of the parental relationship may be due to continuing important social support or due to the influence of parental attachment in terms of ability to form later social and intimate bonds [3, 11]. The current paper provides support for this latter explanation as we show this association persists into even older adulthood. Wiseman et al. (2006) and Jackson (2007) also demonstrated associations between parental care and loneliness as well as parental overprotection and loneliness [2, 4] with Jackson suggesting based on their findings that these associations disappear in university once peers become more important than parents. This was however contrasted by Wiseman who found an association persisting in university students; by Von Soest who as above found an association persisting at age 31; and by the current paper where this associations was present in adults aged 50 and over [2, 3].

The indication that parental bonding in early years impacts the ability to form later social and intimate bonds informed the hypothesis that social isolation could be a potential mediator of the association between parental bonding and loneliness in this sample. However, the findings of the current study suggest that, rather than purely a lack of connections, parental bonding is also associated with less satisfaction with the relationships one does have, given the consistent association of all parental bonding indicators with loneliness independent of social isolation. This is supported by attachment theory which states a dissatisfaction with connections can be linked to parental bonding and attachment [9], and the more recent literature which describes how early experiences of a lack of parental care and nurturance can lead to an impaired self-concept which in turn leads the individual to continually anticipate rejection and criticism thus leading to both less socialising as well as dissatisfaction with the interpersonal relationships one does have and as a result loneliness [2, 26]. An association between persistent loneliness and dissatisfaction with the relationships one does have has also been previously noted in college students [27]. Further research will be needed to replicate these associations in older adults as well as to further illustrate the pathways through which parental bonding may impact loneliness in later life including the role of personality.

The associations found in the current study while consistent were not large. It would be valuable to observe associations in relation to chronic loneliness over the life course once such data becomes available. The current study allowed us to look at recurrence of loneliness over a 8-year period (waves 4–8) and associations were indeed stronger for loneliness at multiple waves compared to single wave loneliness. Recent research highlights that parental bonding and issues with same may be of particular importance in relation to chronic or consistent life course loneliness in particular [28] and this is therefore worth exploring quantitatively in population-based datasets. The stronger associations found in the current paper for multiple wave loneliness do suggest that the influence of parental bonding is linked to more chronic type loneliness though even within this 8-year period and therefore again highlights the need for a range of interventions for a range of root causes of loneliness [29, 30] with this group likely needing interventions more focused on addressing maladaptive social cognitions or thought patterns [31] such as cognitive behaviour therapies, rather than situational factors [28].

### Strengths

Strengths of this study include the large population-based sample of adults aged 50 and over provided by ELSA which provided nationally representative data as well as the power to adjust for multiple confounders. The large representative sample also means results of this analysis can be generalised to the population. The longitudinal nature of the data meant it was possible to observe associations over time and look at both chronic isolation and loneliness as well as presence at a single wave. By looking at the potential role of isolation as a mediator in order to build on theories explaining the role of parental bonding in loneliness as well as exploring these associations in an older population this paper addressed important gaps in the literature to date. Finally, the use of previously used and validated measures to assess loneliness [16, 17] isolation [18, 22, 23] and parental bonding [20, 21] was an additional strength.

### Limitations

There are a number of key limitations to consider in relation to the current study. The reliance on self-report data means that the introduction of bias must be considered particularly in relation to recall bias and attribution error given data on parental bonding was based on later life recall. For instance, it has previously been demonstrated that those suffering from depression recall the past more negatively [32] and the same may occur in relation to loneliness which is also in any case a strong predictor of depression [33]. It is possible that those who are more lonely now recall their parenting experiences as worse, rather than worse experiences simply leading to later loneliness. This was however somewhat tempered by looking at associations with isolation and loneliness at later waves only. Further longitudinal research will be required to clarify direction of effects in relation to parental bonds and later loneliness.

Additionally, while the majority of parental indicators showed no significant association with missing follow up data for loneliness, two small associations were found [Appendix i] and although the effect sizes remained small it is important to note the presence of these associations and future longitudinal analyses needs to continue to assess for attrition associated with key variables.

Findings will also need to replicated in other cultures beyond the English sample employed in this analysis. Finally, it may be important to also consider generational differences in relation to parental bonding and how scores might compare for older versus younger cohorts in further research on links with loneliness.

## Conclusion

The results of the current study show the consistent significant associations between indicators of parental bonding and loneliness persist into older age. The importance of both care and overprotection as well as the influence of both maternal and paternal bonds was shown. Parental bonding was also associated with later life isolation in this population-based sample of adults aged 50 and over although the association with loneliness was independent of isolation. Further research is now needed to further illustrate the pathways by which parental bonding may impact later life loneliness and isolation in order to best inform both intervention and prevention in relation to both services and policy.

## Supplementary Information

**Additional file 1.** Appendix I: Missing data.

## Data Availability

The English Longitudinal Study of Ageing dataset is publicly available via the UK Data Service [http://www.ukdataservice.ac.uk].
