# Supplementary material for: Associations between parental bonding, social isolation and loneliness: do associations persist in later life and is isolation a mediator between parental bonding and loneliness?
Source: BMC Psychol. 2022 Jun 16;10:152. doi: 10.1186/s40359-022-00855-z (PMC9202109; doi:10.1186/s40359-022-00855-z)
Supplement: Supplementary file 1 — Additional file 1. Appendix I: Missing data. [file 40359_2022_855_MOESM1_ESM.docx]

## Appendix I

T-tests for significance were run to check for associations between parental bonding indicators and missing data for isolation and loneliness. No significant associations were found in relation to isolation data. In relation to loneliness data, no significant associations were found in relation to 7 of the 9 indicators. However, overall PBI scores for fathers specifically, were significantly associated with missing data for loneliness at waves 4-8 with slightly lower scores for those missing data [m=21.7 sd 3.38] compared to those not missing data [M=22 SD 3.28] [t=2.22 p=.03]. Overall overprotection scores were also slightly higher [M=15.6 SD 3.32] in those missing loneliness data compared to those not missing follow up data [M 15.2 SD 3.40][t=-2.45 p=.014].
